# Genetic selection modulates feeding behavior of group-housed pigs exposed to daily cyclic high ambient temperatures

**DOI:** 10.1371/journal.pone.0258904

**Published:** 2022-01-24

**Authors:** Alícia Zem Fraga, Luciano Hauschild, Paulo Henrique Reis Furtado Campos, Marcio Valk, Débora Zava Bello, Marcos Kipper, Ines Andretta

**Affiliations:** 1 Department of Animal Science, School of Agricultural and Veterinarian Sciences, São Paulo State University, Jaboticabal, São Paulo, Brazil; 2 PEGASE, INRAE, Institut Agro, Saint Gilles, France; 3 Department of Animal Science, Universidade Federal de Viçosa, Viçosa, Minas Gerais, Brazil; 4 Department of Statistics, Universidade Federal do Rio Grande do Sul, Porto Alegre, Rio Grande do Sul, Brazil; 5 Department of Animal Science, Universidade Federal do Rio Grande do Sul, Porto Alegre, Rio Grande do Sul, Brazil; Tokat Gaziosmanpasa Universitesi, TURKEY

## Abstract

This study was conducted to evaluate the effect of genetic selection (Lines A and B; Line A pigs have a greater proportion of Pietrain genes than those from Line B and therefore, selected for improved lean tissue accretion) on the feeding behavior of group-housed pigs exposed to daily cyclic high ambient temperatures. Feeding behavior of 78 barrows housed together in a single room was recorded in real time by five automatic feeders. The feeders registered each visit of each pig (day, hour, min, and second) and the amount of feed requested. Daily cyclic high ambient temperature was induced exposing pigs at 22°C from 18.00 to 10.00 h and 30°C from 10.01 to 17.59 h. From this temperature variation, day-period was divided into: 22°C_(06-10h)_, from 6.00 to 10.00 h; 30°C_(10-18h)_, from 10.01 to 17.59 h; and 22°C_(18-06h)_, from 18.00 to 5.59 h. Meal criteria was estimated based on the probability of animals starting a new feeding event within the next minute since the last visit (Pstart). After defining the meal criteria, the number of meals (n), feed intake rate (g/min), feed intake (g/meal), feeder occupancy (min/meal), and interval between meals (min) of each animal were calculated. Greatest probability of starting to feed was observed at 22°C_(06-10h)_, followed by 30°C_(10-18h)_ and then 22°C_(18-06h)_. Regardless of time period, pigs from line A had greater feed intake rate and lower feed intake, feed occupancy per meal and probability of starting a meal when compared with line B pigs. Only line A pigs had greater feed intake and feeder occupancy per meal at 22°C_(18-06h)_ than remainder of the day. This indicates that pig feeding pattern is strongly related to the circadian rhythm. However, the genetic selection for improved lean tissue accretion may modulate pigs feeding behavior under daily cyclic high ambient temperatures.

## Introduction

Pigs show a diurnal feeding pattern characterized by two peaks of feeding activity over 24 h-day [1]. However, the feeding behavior of pigs may be affected by several factors, including genetic selection [2] and environmental conditions [3]. Mainly through changes in meal size, lower feed intake for pigs under cyclic high ambient temperature has been observed when compared to thermoneutral conditions [4]. Despite such scientific finding, most of the information of heat stress on feeding behavior of pigs were obtained from experimental protocols with animals housed individually (or with few animals per pen), and/or exposed to constant high temperatures during the day [5]. Such studies do not apply to commercial conditions, especially in tropical areas, in which group-housed pigs are usually reared in semi-open buildings and then exposed to daily temperature variations. In addition, animals selected for improved lean tissue accretion produce more metabolic heat and may have a reduced capacity to cope with heat stress [6].

A better understanding of feeding behavior may contribute to the genetic selection of individuals based on their thermoregulatory responses, productive traits, and well-being. Furthermore, the detection of changes in feeding behavior may be an interesting and useful tool for identifying heat stress in the early stages of exposure and may help in decision-making to prevent major losses. Equipment such as electronic feeders provides a promising approach to evaluate pigs feeding behavior responses since it allows recording automatically information on feed intake of individual pigs in a group-housed condition [7]. In a previous study, we have demonstrated that pigs selected for improved lean tissue accretion (i.e., with a greater proportion of Pietrain genes) had lower growth performance when housed under daily cyclic high ambient temperature conditions [8]. We hypothesized that in such conditions, pigs prioritize feed intake in the coolest hours of the day and that their feeding behavior is modulated by genotype. Therefore, in the current study we further evaluated the effects of genetic selection on the feeding behavior of group-housed growing-finishing pigs exposed to daily cyclic high ambient temperatures.

## Material and methods

All experimental procedures were reviewed and approved according to the current ethical standards of the Ethical Committee for Care and Use of Experimental Animals of the School of Agricultural and Veterinarian Sciences of São Paulo State University (protocol N° 18077/16).

### Data origin

Pigs feeding database was obtained from our previous study [8]. In this study, the feeding behavior of 78 barrows (22 ± 2.5 kg of body weight, BW) from two commercial genetic lines (39 pigs of each genetic line, reported hereafter as Line A and Line B; Agroceres PIC, Rio Claro, Brazil) was recorded in real-time by five automatic feeders (AIPF; University of Lleida, Lleida, Spain). Both lines consisted of Large White × Landrace × Duroc × Pietrain multiple crosses, with Line A pigs having a greater proportion of Pietrain genes than those from Line B, as previously described [9]. Pigs were housed together in a single room (1.19 m^2^/animal; without environmental enrichments) with a full concrete floor equipped with an evaporative pad cooling system (Big Dutchman, Araraquara, SP, Brazil) and electric heaters, automatically controlled to maintain the ambient temperature at 22°C from 18.00 to 10.00 h and 30°C from 10.01 to 17.59 h. These temperatures aimed to simulate daily cyclic ambient temperature variations that pigs are usually exposed to in tropical climate areas.

Pigs remained in the experiment for 102 days, which consisted of an 18-day adaptation period and a subsequent 84-day experimental period, divided into growing phase 1 (30 to 45 kg BW; days 0 to 20); growing phase 2 (45 to 75 kg BW; days 21 to 48); and finishing phase (75 to 105 kg BW; days 49 to 83). Pigs had free access to water delivered via 10 beat ball drinkers distributed all over the pen and were fed *ad libitum* with diets formulated to meet their nutritional requirements of the animals with the highest potential for lean deposition (Line A) according to NRC [10] recommendations. The photoperiod was fixed from 6.00 to 18.00 h of artificial light controlled by a timer switch. At the beginning of the adaptation period, one transponder (plastic button tag containing passive transponders of radio frequency identification; Allflex, Joinville, SC, Brazil) was inserted in the right ear of each pig using tagger pliers, and then animals were introduced to the electronic feeders. The use of exclusive identification codes per pig allowed recording individual feed intake over the trial with all animals housed in the same pen in a single group.

The functioning of these feeders was previously described [11]. The electronic feeders (AIPF) identify each pig when its head is introduced into the feeder and deliver feed in response to each animal request. The serving size was 15 g at the beginning and 25 g at the end of the experiment. A time lag (18 sec) was imposed to ensure that pigs consumed each serving before requesting a new one. Pigs tended to leave the feeder hopper empty or to leave small amounts of feed after each visit. All pigs in the pen could access any of the feeders.

### Overview of data compilation

Feed intake was calculated using the feeding information of each pig (AIPF Software). The feeders were equipped with a monitoring tool that continuously registered each visit of each pig with start and stop times (day, hour, minute, and second) and the amount of feed requested. Feeding information collected on days on which animals were handled (weighed or scanned, six days) or on days that some eventuality happened (equipment malfunctions, momentaneous data record error, etc., which correspond to 12 days) were removed from the data. The total information from the initial data set consisted of 129,167 records, which were then analyzed using R Statistical Software (version 2.14.0; R Foundation, Austria).

The presence of outliers was evaluated through graphical analyses and by daily records of anomalies during the experimental period. Therefore, the first procedure was data filtering. Data of animals whose interval between visits was greater than 720 min (3.78% of observations from the initial database) were arbitrarily excluded from the database and assumed to be physiologically noncompatible data [12]. After a graphical analysis of the new database, a second filter criterion was applied. Data of feeder occupancy greater than 30 min (0.48% of observations) and zero feed intake (0.35% of observations) were also excluded. Records without feed intake may be associated with animal-feeder interactions, in which quick entry and exit of animals at the feeder are recognized by the software. Therefore, data filtering, although arbitrary and variable according to the database, is a biologically relevant step since it allows a more realistic view and avoids possible incorrect conclusions garnered from the analysis of feeding behavior. After the filtering steps, no other discrepancies were observed in the database (more details can be found in S1 Dataset).

### Meal criteria

A pig visit to the feeder is not necessarily a meal. As aforementioned, a visit record can be caused by the quick entry and exit of the animal, which may even be considered a nonfeeding event. Therefore, expressing feeding behavior data in terms of meals is considered a more appropriate unit [13]. A meal is defined as a cluster of feeding visits interrupted by short pauses [14]. Then, a criterion clustering visits into a single meal must be applied. This point is important for studies of animal behavior since the absence of the definition of a meal criterion (or even the mathematical method to be used) may result in the loss of biologically significant information. For this purpose, the probability of animals starting a new feeding event within the next minute since the last visit (Pstart) was calculated for each factor of our study (i.e., periods of the day and genetic lines). Thus, the meal criteria were estimated as the first point that minimizes Pstart curve as was previously described [15]. To reduce the effect of random variation in the Pstart function, a simple moving average (SMA) over 5-min intervals was used.

### Calculations and statistical analysis

After defining the meal criteria, a new database was created using the meal criterion to group visits that have composed the same meal. The new database consisted of 39,680 records. This database was used to calculate the number of meals (n), feed intake rate (defined as the feed intake for each minute spent feeding, g/min), feed intake (g/meal), feeder occupancy (min/meal), and the interval between meals (min) of each animal. As pig feeding behavior changes throughout the 24-h day [1], for a more adequate approach and considering temperature variation during the day, the day was divided into three periods: 22°C_(06-10h)_, from 6.00 to 10.00 h; 30°C_(10-18h)_, from 10.01 to 17.59 h; and 22°C_(18-06h)_, from 18.00 to 5.59 h. Because the periods of the day had different durations, feeding behavior variables were expressed per hour.

The Cramer-von Mises test was used to verify the normality of studentized residuals. The data were analyzed using the linear mixed effects model framework. The implementation was via the MIXED procedure of SAS (version 9.3, SAS Inst. Inc., Cary, NC) and were presented hereafter as least squares means. The individual pig was considered the experimental unit and the initial BW (weight of the animal on the first day of the experimental period) was used as covariable between genetic lines when significant in the model. The model included the fixed effects of the period of the day (TP), genetic line (GL), experimental phase (P), and their interactions (TP×GL, TP×P, GL×P, and TP×GL×P) as follow:

Yijmk=μ+αi+βj+γm+(αβ)ij+(αγ)im+(βγ)jm+(αβγ)ijm+eijmk

where Yijmk is the observed variable (observation for the *k*th animal from the (i,j,m)th cell), μ is the overall mean, αi is the effect of the *i*th level of the TP factor, βj is the effect of the *j*th level of the GL factor, γm is the effect of the *m*th level of the P factor, and their interactions (that is, *i*th level of TP with the *j*th level of GL, (αβ)ij; *i*th level of TP with the *m*th level of P, (αγ)im; *j*th level of GL with the *m*th level of the P, (βγ)jm; and *i*th level of TP with the *j*th level of GL with the *m*th level of the P, (αβγ)ijm). The e_*ijmk*_ was considered the random error of *k*th observation from (i,j,m)th. The repeated measurement option was used with a compound symmetry covariance structure to account for the animal effect over sampling time. Differences were considered significant if *p* < 0.05. When there was an effect of an interaction between period and genetic line (*p* < 0.05), adjusted means of genetic lines were compared in each period using the Tukey-Kramer test.

### Prandial correlations

The prandial correlations were calculated for each animal individually through Pearson’s correlation between the variables “meal size, min” and “interval between meals, min”. The correlation between “meal size” and “interval before meal” (pre-prandial) indicates satiety mechanism; whereas the correlation between “meal size” and “interval after meal” (post-prandial) indicates hunger mechanism [16]. The pigs were classified in four groups according to the significant correlation coefficients (*p* < 0.05) that showed: pre-prandial, post-prandial, pre- and post-prandial regulations, or no prandial correlations when both were not significant.

## Results

### General observations

Room ambient temperature averaged 22.3 ± 0.4°C from 18.00 to 10.00 h and 30.2 ± 0.5°C from 10.01 to 17.59 h. The daily relative humidity averaged 70.35 ± 3.20%. These values were in accordance with our predefined objectives. Performance data were reported in our previous study [8], and individual pig behavior profiles are available in S1 Fig. Briefly, irrespective of the growth phase, pigs from genetic line A had lower average daily feed intake (ADFI), average daily gain (ADG), and final BW than pigs from line B (*p* < 0.01). At the end of the experiment, the difference in BW between lines was 11 kg on average (112.15 and 129.58 kg for line A and line B pigs, respectively; *p* < 0.01).

### Pstart and meal criteria

**Fig 1:**
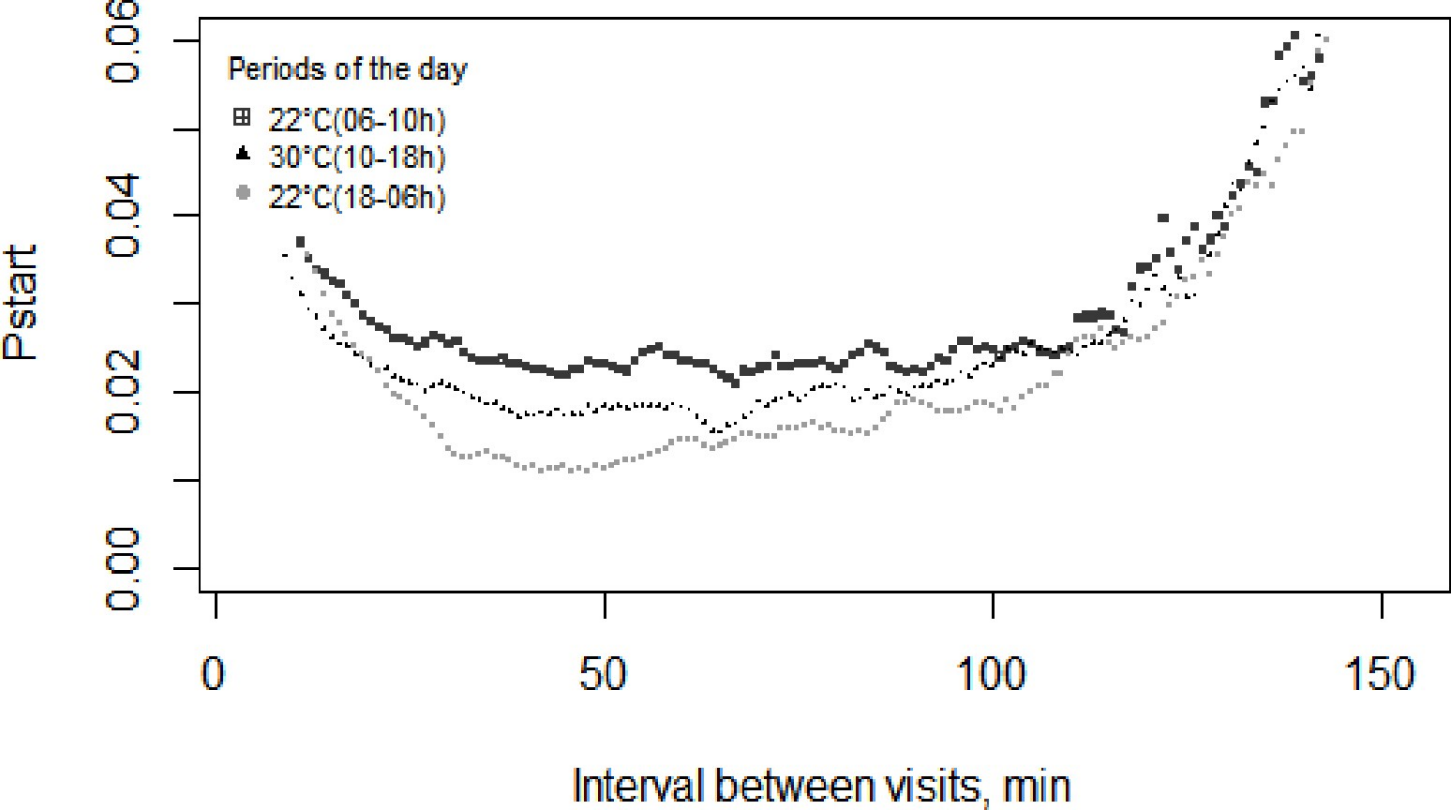
Probability of growing-finishing pigs exposed to daily cyclic high ambient temperature starting a new feeding event within the next minute since the last visit (Pstart) at 22°C_(06-10h)_, 30°C_(10-18h)_, and 22°C_(18-06h)_.

**Fig 2:**
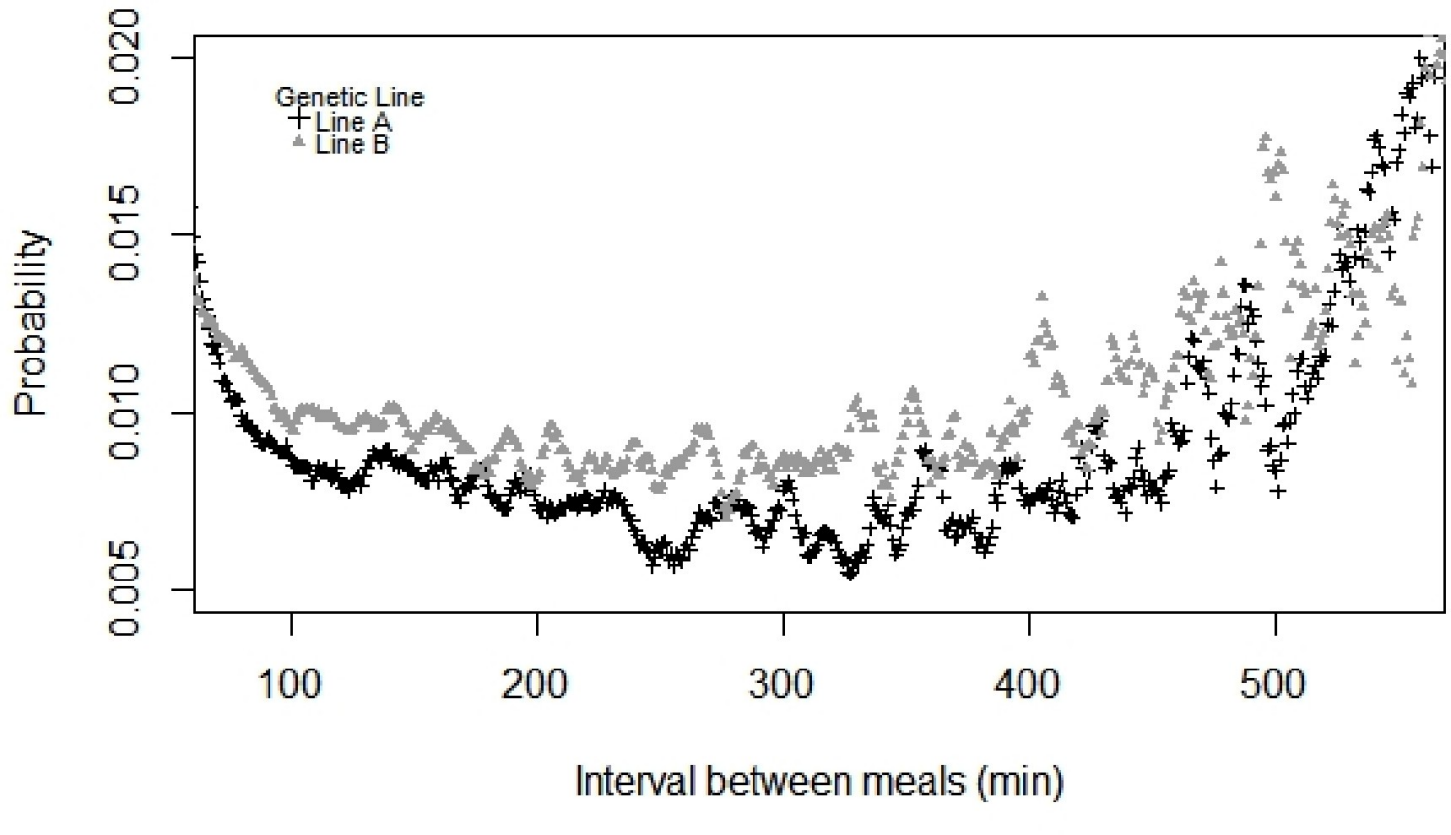
Probability of growing-finishing pigs exposed to daily cyclic high ambient temperature starting a meal according to the interval between meals (min) considering two genetic lines. Genetic Line A pigs had greater proportion of Pietrain genes than those from genetic Line B pigs.

Among the three periods, Pstart difference was observed up to approximately 100 min, a period in which the greatest probability of starting to feed was observed at 22°C_(06-10h)_, followed by 30°C_(10-18h)_ and 22°C_(18-06h)_ (Fig 1). The meal criteria values were 48 min at 22°C_(06-10h)_, 47 min at 30°C_(10-18h)_ and 49 min at 22°C_(18-06h)_; and for line A and line B were 52 min and 46 min, respectively. Therefore, as we had different periods (n = 3) and genetic lines (n = 2), the meal criteria applied in our database was the average of these five results (48 min). After defining the meal criteria, differences between the genetic lines (with a lower probability of line A to start a meal compared with line B) were observed mainly up to 450 min (Fig 2).

### Feeding behavior responses

#### Exploratory data analysis

Feeding behavior responses of growing-finishing pigs are presented in Table 1. Residuals of all dependent variables were normally distributed, and initial BW as covariate was significant (*p* < 0.01) for all feeding behavior variables. All responses were influenced by period, genetic line, and experimental phase (*p* < 0.01).

**Table 1:** Feeding behavior according to periods of the day and genetic lines of growing-finishing pigs exposed to daily cyclic high ambient temperature during 84-days of the experimental period^1^^,^^2^.

Periods	22°C_(06-10h)_		30°C_(10-18h)_		22°C_(18-06h)_		Statistical analysis (*p*-value)^4^			
Genetic Lines^3^	A	B	A	B	A	B				
Number of animals	39	39	39	39	39	39	TP×GL	TP×P	GL×P	TP×GL×P
Feed intake rate, g/min	27.71 (0.5)	26.86 (0.8)	27.11 (1.2)	26.00 (2)	25.38 (0.5)	24.93 (2)	0.40	0.13	<0.01	0.98
Feed intake, g/meal	182.25^c^ (8)	256.46^b^ (12)	241.67^b^ (15)	299.91^a^ (9)	306.33^a^ (3)	267.74^b^ (4)	<0.01	<0.01	<0.01	0.17
Feeder occupancy, min/meal	6.96^e^ (3)	9.78^c^^d^ (3)	9.29^d^ (4)	11.69^a^^b^ (0.5)	12.43^a^ (0.4)	10.81^b^^c^ (2)	<0.01	0.01	<0.01	0.61
Interval between meals, min	162.19^b^^c^ (7)	128.60^d^ (4)	180.21^a^ (12)	150.14^c^ (5)	184.06^a^ (9)	173.22^a^^b^ (4)	0.02	0.56	0.08	0.39
Number of meals^5^, n/pig	0.29 (0.1)	0.49 (0.1)	0.28 (0.4)	0.38 (0.3)	0.21 (0.2)	0.22 (0.2)	<0.01	<0.01	0.09	0.01

^1^Data after application of meal criteria defined as 48 min and expressed per hour of the day since the periods had different duration (22°C_(06-10h)_: from 6.00 to 10.00 h, 30°C_(10-18h)_: from 10.01 to 17.59 h; 22°C_(18-06h)_: from 18.00 h to 5.59 h).

^2^Least squares means and standard error (SE).

^3^Genetic line A pigs had a greater proportion of Pietrain genes than those from genetic line B pigs.

^4^Probability of periods of the day (n = 3; TP), genetic line (n = 2; GL), experimental phase (n = 3; P), and interactions between TP×GL, TP×P, GL×P, and TP×GL×P. There was an effect of TP, GL, and P (*p* < 0.01) for all variables studied. For variables whose TP×GL interaction was significant, average values were analyzed by Tukey’ test. Initial BW as a covariate was significant for all variables; *p* < 0.01.

^5^The TP×GL×P interaction for the number of meals is presented in Table 2.

^a,b,c^ Values within a row with different superscripts differed between genetic lines in each period of the day (*p* < 0.05).

Regardless of line and experimental phase, feed intake rate was greater at 22°C_(06-10h)_, followed by 30°C_(10-18h)_, and then 22°C_(18-06h)_ (*p* < 0.05; Table 1). A GL×P interaction was observed for feed intake rate (*p* < 0.01; Table 1). Regardless of time period, line A pigs had greater feed intake rate than line B pigs in growing phase 2 (26.53 vs 24.98 g/min; *p* < 0.01) and total period (26.73 vs 25.95 g/min; *p* = 0.01).

Interactions between TP×GL, TP×P, and GL×P were significant for feed intake and feeder occupancy per meal (Table 1). At 22°C_(06-10h)_ and 30°C_(10-18h)_, pigs from line A had lower feed intake and feeder occupancy per meal than pigs from line B (*p* < 0.05; TP×GL). In contrast, at 22°C_(18-06h)_, line A pigs had greater feed intake and feeder occupancy per meal than line B pigs (*p* < 0.05). Despite of no significant differences among periods in growing phase 1, in the following phases, feed intake and feeder occupancy were decreased at 22°C_(06-10h)_ compared with 30°C_(10-18h)_ and 22°C_(18-06h)_ (*p* < 0.05; TP×P). Besides, except in growing phase 1, in which feed intake and feeder occupancy per meal did not differ between lines, both were lower for line A than line B pigs in the subsequent phases (*p* < 0.05; GL×P).

A TP×GL interaction was observed for the interval between meals (*p* = 0.02; Table 1). At 22°C_(06-10h)_ and 30°C_(10-18h)_, pigs from line A had greater interval between meals than pigs from line B (*p* < 0.05). At 22°C_(18-06h)_, no difference between lines was found (*p* = 0.34). For both lines, regardless of period, the increase in BW between stages of growth was associated with an increased feed intake rate, feed intake per meal, and interval between meals (*p* < 0.05).

Interaction between TP×GL×P was significant for number of meals (Table 1). Overall, at 22°C_(06-10h)_ and 30°C_(10–18 h)_, pigs from line A had lower number of meals than pigs from line B (*p* < 0.05; Table 2). Besides, an increase in the number of meals was observed only in the early stages (from growing phase 1 to growing phase 2), at 22°C_(06-10h)_ for pigs from line A, and at 22°C_(06-10h)_ and 30°C_(10-18h)_ for pigs from line B (*p* < 0.05; Table 2). Only line B pigs became more diurnal throughout the trial, with 77, 79, and 83% of the meals consumed during the light period (at 22°C_(06-10h)_ and 30°C_(10-18h)_) in growing phases 1 and 2 and the finishing phase, respectively.

**Table 2:** Number of meals (n/pig) according to periods of the day, genetic lines, and experimental phases of growing-finishing pigs exposed to daily cyclic high ambient temperature^1^^,^^2^.

Periods	22°C_(06-10h)_		*p*-value^4^	30°C_(10-18h)_		*p*-value^5^	22°C_(18-06h)_		*p*-value^6^
Genetic Lines^3^	A	B	A vs B	A	B	A vs B	A	B	A vs B
Number of animals	39	39		39	39		39	39	
Growing phase 1 (21 days)									
Number of meals, n/pig	0.21* (0.02)	0.34^#^ (0.03)	<0.01	0.23 (0.02)	0.29^#^ (0.02)	0.85	0.15 (0.01)	0.19 (0.05)	0.98
Growing phase 2 (28 days)									
Number of meals, n/pig	0.32* (0.01)	0.57^#^ (0.01)	<0.01	0.31 (0.03)	0.42^#^ (0.05)	<0.01	0.25 (0.02)	0.26 (0.01)	0.99
Finishing phase (35 days)									
Number of meals, n/pig	0.35 (0.01)	0.55 (0.02)	<0.01	0.30 (0.03)	0.44 (0.01)	<0.01	0.24 (0.01)	0.21 (0.05)	0.99
Total period (84 days)									
Number of meals, n/pig	0.29 (0.02)	0.49 (0.02)	<0.01	0.28 (0.01)	0.38 (0.02)	<0.01	0.21 (0.01)	0.22 (0.01)	0.99

^1^Data after application of meal criteria defined as 48 min and expressed per hour of the day since the periods had different duration (22°C_(06-10h)_: from 6.00 to 10.00 h, 30°C_(10-18h)_: from 10.01 to 17.59 h; 22°C_(18-06h)_: from 18.00 to 5.59 h). Additional information regarding the pig feeding behavior profile for each experimental phase is available in S3 Fig.

^2^Least squares means and standard error (SE).

^3^Genetic line A pigs had a greater proportion of Pietrain genes than those from genetic line B pigs.

^4,5,6^Statistical analysis between genetic lines in each experimental phase for 22°C_(06-10h)_^3^, 30°C_(10-18h)_^4^, and 22°C_(18-06h)_^5^.

*,^#^Means differed between experimental phases for pigs from line A* and line B^#^ (*p* < 0.05).

#### Descriptive statistics

**Fig 3:**
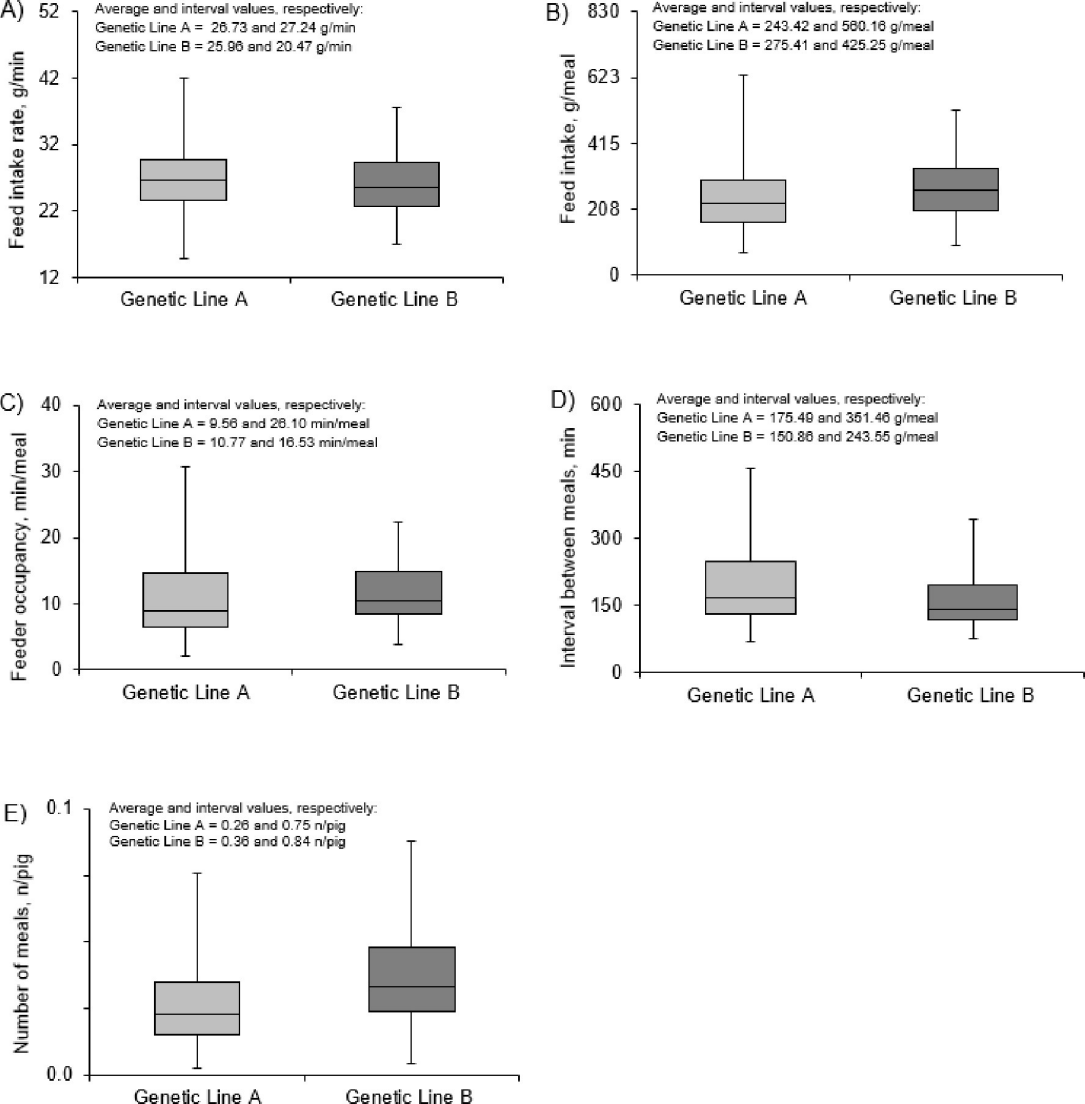
Description statistics of feeding behavior responses for two lines^1^ of growing-finishing pigs exposed to daily cyclic high ambient temperature during 84-days of the experimental period^2,3^. ^1^Genetic Line A pigs had a greater proportion of Pietrain genes than those from genetic Line B pigs. ^2^Data after application of meal criteria defined as 48 min. Such data refer to the total experimental period, regardless of the experimental phase. ^3^Interval value: Difference between the minimum and maximum value.

**Fig 4:**
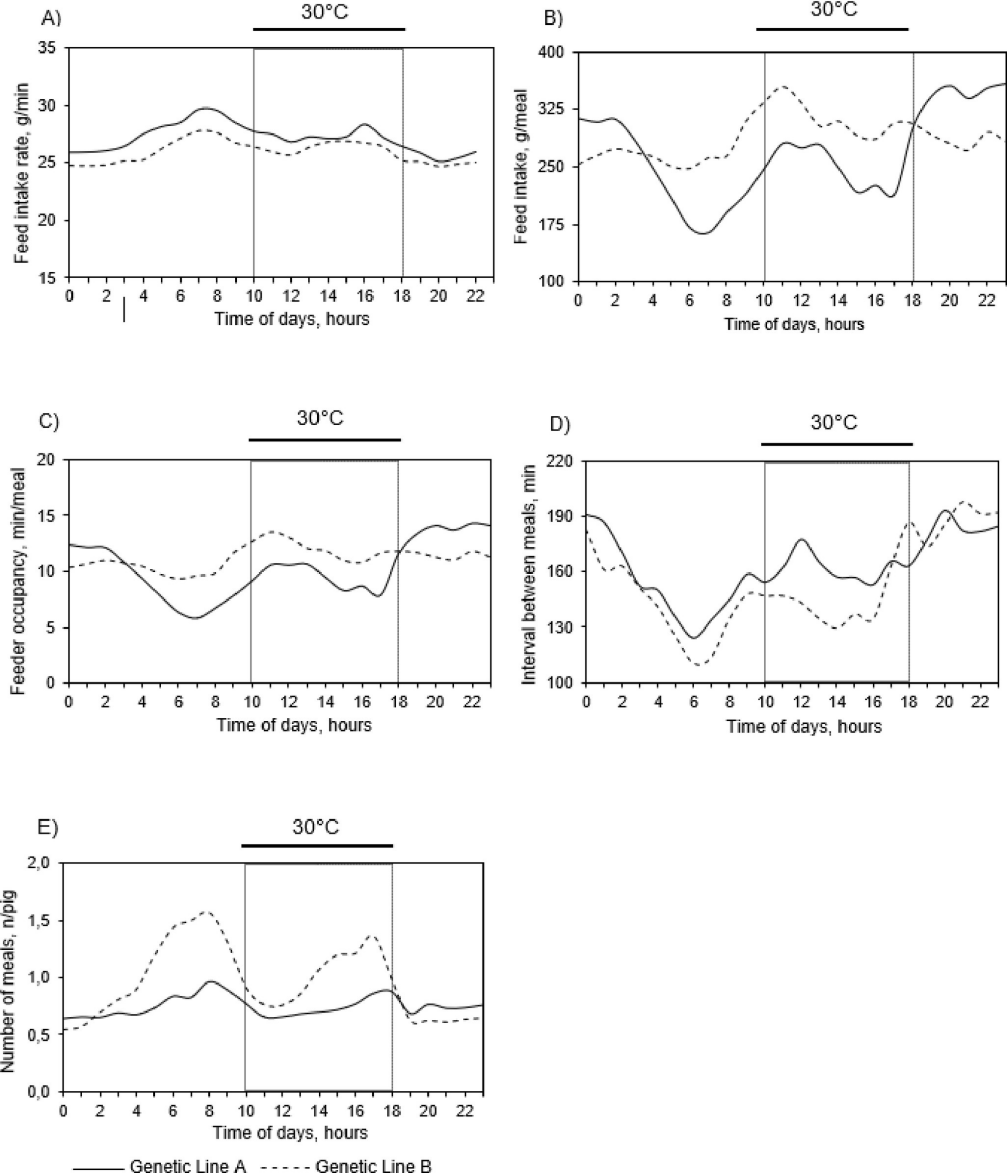
Circadian variation of A) Feed intake rate, B) Feed intake per meal, C) Feeder occupancy per meal, D) Interval between meals, and E) Number of meals for two lines^1^ of growing-finishing pigs exposed to daily cyclic high ambient temperature during 84-days of the experimental period^2^. ^1^Genetic Line A pigs (_______) had greater proportion of Pietrain genes than those from genetic Line B pigs (-—-—-). ^2^ Average data of total experimental period throughout 24 h-day, regardless of the experimental phase.

Descriptive statistics for the total experimental period (from 0 to 84 days) are presented in Fig 3. For feed intake rate, regardless of the experimental phase, the interval values were 27.24 and 20.47 g/min for pigs from lines A and B, respectively (Fig 3). Despite greater dispersion data for line A pigs, the feed intake rate followed a similar pattern throughout the 24h-day for both lines (Fig 4A).

Average values of feed intake and feeder occupancy per meal were lower for line A than line B pigs (Fig 3). Such results were associated with a peak in feed intake and feeder occupancy per meal at 30°C_(10-18h)_ for line B pigs (at 11.00 h; Fig 4B and 4C) and a 14% difference in diurnal feed consumption between lines. In contrast, at 22°C_(18-06h)_ (specifically from 19.00 to 3.00 h), line A pigs had greater feed intake and feeder occupancy per meal than line B (Fig 4B and 4C; *p* < 0.05).

Pigs from line A had greater average value of interval between meals when compared with pigs from line B (175.49 vs 150.86 g/meal; Fig 3). In particular, greater values of interval between meals for line A pigs were observed mainly during the diurnal period (from 12.00 to 16.00h, Fig 4D). Differences between lines with a lower number of meals in line A than line B pigs were observed for all descriptive statistics (Fig 3). This effect was associated with two peaks in the number of meals at approximately 8.00 h and 17.00 h for line B pigs (Fig 4E).

### Prandial correlations

In our study, 49% of group-housed pigs (17 pigs from line A and 21 from line B) showed both pre- and post-prandial regulation. In addition, 21% of pigs (13 pigs from line A and 3 from line B) showed no prandial correlation, 24% (7 pigs from line A and 12 from line B) showed only a post-prandial correlation, and the remaining 6% of the group (2 pigs from line A and 3 from line B) showed only a pre-prandial correlation. Therefore, except for the “no prandial correlations” group, in which the number of animals from line A was numerically greater than that from line B, little difference between lines was observed.

## Discussion

Feeding data from two pig genetic lines with different proportions of Pietrain genes were analyzed to evaluate the genetic selection effect on the feeding behavior of growing-finishing pigs under daily cyclic high ambient temperature conditions. Our major and original finding is that differences in feeding behavior between lines reared under daily ambient temperature variations may partially explain differences in growth performance, which was previously reported [8]. The greatest probability of animals starting to feed was found at 22°C_(06-10h)_, followed by 30°C_(10-18h)_ and then 22°C_(18–06 h)_, suggesting pigs feeding patterns are strongly related to the circadian rhythm. In addition, because pigs from line A had a lower probability of starting a meal than pigs from line B, differences between “hunger” and “satiety” mechanisms could also be attributed to the genetic line effect on feeding behavior. From these results and assuming that feeding behavior patterns can be evaluated through pre- and post-prandial correlations [16], we discuss whether pigs differently selected for lean mass and presumably with different metabolic heat production and thermoregulatory responses, could present different feeding behavior patterns when exposed to daily cyclic high ambient temperature conditions.

In the current study, the greater probability of pigs starting to feed at 22°C_(06-10h)_ compared with 30°C_(10-18h)_ and 22°C_(18-06h)_ reflects the strong influence of circadian rhythm on feeding patterns and may have induced the concomitant access of pigs to the feeders. As a result, pigs ate faster and had lower feed intake and feeder occupancy per meal at 22°C_(06-10h)_ than the remaining of the day. Indeed, greater feed intake rate was highly associated with a shorter feeder occupation time [2], indicating that pigs with faster feed intake rate spend less time feeding. Accordingly, diurnal feeding activities were reported in group-housed pigs exposed to thermoneutral conditions [1], constant high ambient temperature [17], and cyclic ambient conditions [4]. From these results and considering that catabolic and anabolic status follows a circadian rhythm [18], it can be assumed that the periods of activity and feeding inactivity of pigs change over the 24-h day.

When exposed to thermoneutral [1] and constant high ambient temperature conditions [17], group-housed pigs showed a biphasic feeding pattern, characterized by two peaks of feeding activity over 24-h day (a first peak at the beginning and another at the end of the day). Such feeding behavior (two peaks) is defined mainly by decreased melatonin levels in the morning and cortisol in the afternoon [18]. Despite such scientific evidence, similar results in the current study were observed only for line B pigs, whose number of meals also presented two peaks (at 8.00 h and 17.00 h) and there was a peak in feed intake per meal at 11.00 h. In agreement, two peaks in the number of visits (one at 9.00 h and the other at 15.00 h) and a greater feed intake per visit value (at approximately 18.00 h) was previously reported for pigs under thermoneutral conditions [2]. Furthermore, pigs from line A had a lower number of meals, feed intake, and feeder occupancy per meal compared with line B during the diurnal period (22°C_(06-10h)_ and 30°C_(10-18h)_). Greater diurnal feed intake per meal (mainly between 15.00 h and 18.00 h; [1]) and lowest number of visits to the feeder from 20.00 to 4.00 h [19] were reported for group-housed pigs raised at thermoneutrality. From these results, it can be suggested that only pigs from line B had feeding behavior patterns similar to nonchallenged pigs, evidencing a better capacity of these animals to cope with our experimental conditions. Because the interval between meals is commonly opposite of meal frequency [18], the greater interval between meals was observed for line A pigs at 22°C_(06-10h)_ and 30°C_(10-18h)_.

Regarding 22°C_(18-06h)_, pigs from line A had substantially greater feed intake and feeder occupancy per meal compared with pigs from line B. It should be noted that line A pigs had a higher proportion of Pietrain genes, which can explain their greater potential for lean depositions than line B pigs [8]. Because the energetic cost of proteogenesis is greater than that of lipogenesis [20], increased lean tissue accretion has been related with increased metabolic heat production [21] with consequent greater susceptibility to heat stress [20]. Therefore, the greater heat production of modern lean genotypes in association to their greater protein deposition and growth rate may explain the increased feeding activity of line A pigs during the nocturnal period. In fact, it seems that pigs from line A may have tried to compensate for the lower feeding activity in the diurnal period changing part of their feeding behavior to the night. Adaptive mechanisms such as changes in feeding behavior patterns and decrease of feed intake can help pigs to reduce metabolic heat production and/or increase heat dissipation when exposed to high ambient conditions [17, 22]. From these scientific findings, it can be suggested that line A pigs are more dependent on thermoregulatory responses to maintain homeothermy. The magnitude of these results is in accordance with the pig’s flexibility to modify their feeding patterns when exposed to limiting conditions [23, 24].

In the current study, the greater feed intake rate of line A pigs in growing phase 2 and total period may be understood as an attempt of these animals to meet their nutritional requirements; however, their behavioral activities may have been limited during the trial. Indeed, feed intake rate is an intrinsic characteristic of the individual and positively correlated with feed intake [25], protein retention [26], and feeding motivation [27]. Therefore, results of greater feed intake rate, associated with lower feed intake, feeder occupancy per meal, and probability of line A pigs starting a meal compared with line B pigs, suggest that pigs from line A were presumably more affected by our experimental conditions. In addition, although no difference between lines was reported in growing phase 1 for feed intake and feeder occupancy per meal, in the subsequent phases, both were lower for line A than line B pigs. Such metabolic and behavioral differences between lines may partially explain the contrasting performance results, in which lower values of ADFI, ADG, and final BW were observed for pigs from line A than line B [8]. Besides, feed intake of pigs became more diurnal with increasing BW and/or age as previously reported [1, 28]. Despite such scientific findings, an increase in diurnal feed intake and the number of meals throughout the trial were observed only for line B pigs (in which, 77, 79, and 83% of the meals were performed during light period in growing phases 1 and 2 and finishing phase, respectively). These findings offer compelling evidence that genetic selection for lean growth may result in changes in pigs feeding behavior activity when exposed to daily cyclic high ambient temperatures.

When comparing lines, the number of line A pigs with “no prandial correlations” was greater than that of line B (13 vs 3 pigs). This result demonstrates that some individuals from line A started to feed randomly without necessarily having a stimulus, and such a response may be a consequence of their lower ability to cope with daily cyclic high ambient temperatures conditions. As a result, a greater dispersion of feeding behavior responses (mainly for feed intake rate, feed intake, and feeder occupancy per meal) was observed for line A than line B pigs. The impact of between-animal variation was highlighted in a previous study [5], which reported that characteristics intrinsic to animals such as breed, BW, and physiological status could be an additional source of variation among studies. These findings are in close agreement with the large variability of results that exist between studies considering the high ambient temperature effects.

Regardless of the period and genetic line, the increase in BW throughout the experimental phases was mainly associated with a greater feed intake rate and feed intake per meal, which resulted in an increase in total feed intake. Similarly, an increase in feeding rate, meal size, and total feed intake was observed with an increase in BW [4, 29]. Since the feeding rate increased by approximately 0.47 g/min per kg increase in BW, such result may be a consequence of increase in body size and physical capacity of feed intake [25].

In conclusion, despite our initial hypothesis that pigs could prioritize their feed intake in the coolest hours of the day (i.e., at 22°C_(06-10h)_ and 22°C_(18-06h)_), our results demonstrate that there was a considerable and dynamic genetic selection effect able to modulate the pigs feeding behavior responses when exposed to daily temperature variations. The greatest probability of animals starting to feed found at 22°C_(06-10h)_, followed by 30°C_(10-18h)_, and then 22°C_(18–06 h)_, suggest that pigs feeding pattern is strongly related to the circadian rhythm. Furthermore, the greater feeding activities of line A at 22°C_(18-06h)_ than the remaining of the day supports the pigs’ capacity to modify their feeding activity under daily cyclic high ambient temperatures. Since our results showed that genetic selection affects feeding behavior under daily cyclic high ambient temperature conditions, research on genetic selection for thermotolerant animals is encouraged.

## Supporting information

S1 Fig Individual pigs behavior profile during the total experimental period (days 0 to 83) throughout 24 h-day. (PDF)

S2 Fig Pigs feeding behavior profile in the growing phase 1 (days 0 to 20), growing phase 2 (days 21 to 48), finishing phase (days 49 to 83), and total experiment period (days 0 to 83). Average data throughout 24 h-day for each genetic line are presented separately.
(PDF)

S3 Fig (DOCX)

S1 Dataset Data filtering diagram. (PDF)
