# Supplementary material for: Genetic selection modulates feeding behavior of group-housed pigs exposed to daily cyclic high ambient temperatures
Source: PLoS One. 2022 Jan 24;17(1):e0258904. doi: 10.1371/journal.pone.0258904 (PMC8786115; doi:10.1371/journal.pone.0258904)
Supplement: S1 Fig — (PDF) [file pone.0258904.s001.pdf]

S1 Fig. Individual pigs behavior profile during the total experimental period (days 0 to 83) throughout 24 h-day.

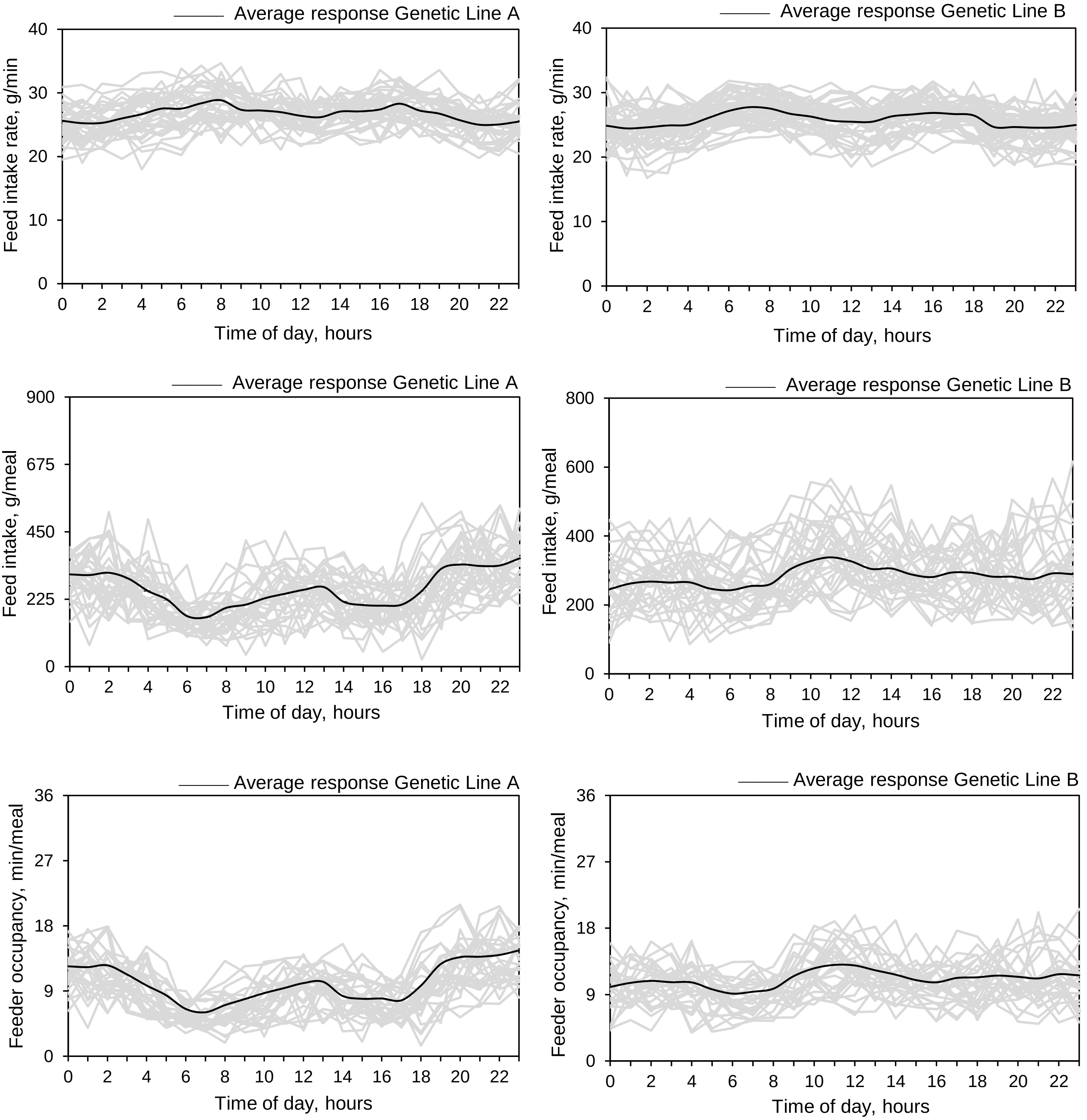

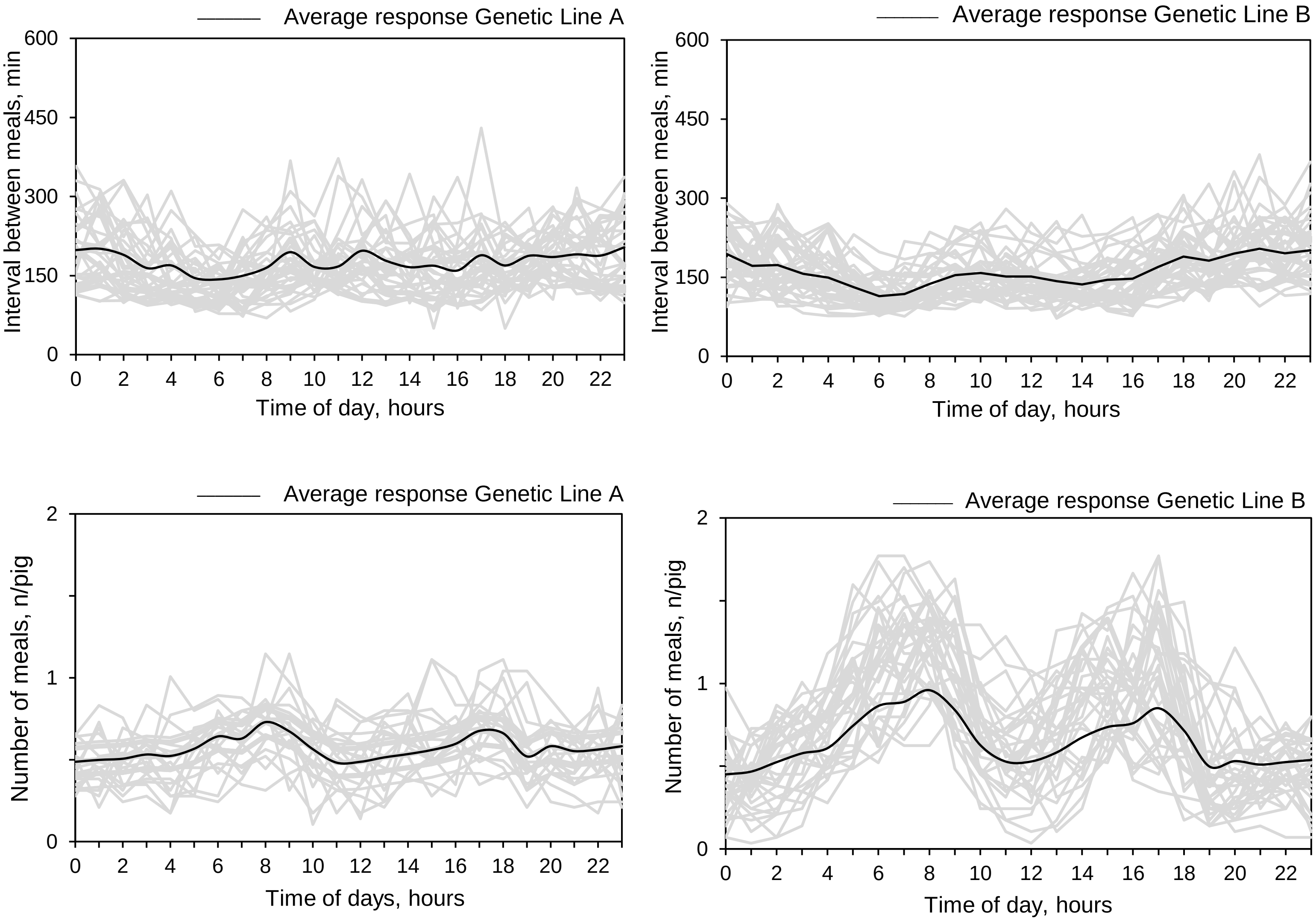
Average response Genetic Line A
Interval between meals, min
Time of day, hours
Average response Genetic Line B
Interval between meals, min
Time of day, hours
Average response Genetic Line A
Number of meals, n/pig
Time of days, hours
Average response Genetic Line B
Number of meals, n/pig
Time of day, hours
