# Supplementary material for: Genetic selection modulates feeding behavior of group-housed pigs exposed to daily cyclic high ambient temperatures
Source: PLoS One. 2022 Jan 24;17(1):e0258904. doi: 10.1371/journal.pone.0258904 (PMC8786115; doi:10.1371/journal.pone.0258904)
Supplement: S2 Fig — Average data throughout 24 h-day for each genetic line are presented separately. (PDF) [file pone.0258904.s002.pdf]

S2 Fig. Pigs feeding behavior profile in the growing phase 1 (days 0 to 20), growing phase 2 (days 21 to 48), finishing phase (days 49 to 83), and total experiment period (days 0 to 83). Average data throughout 24 h-day for each genetic line are presented separately.

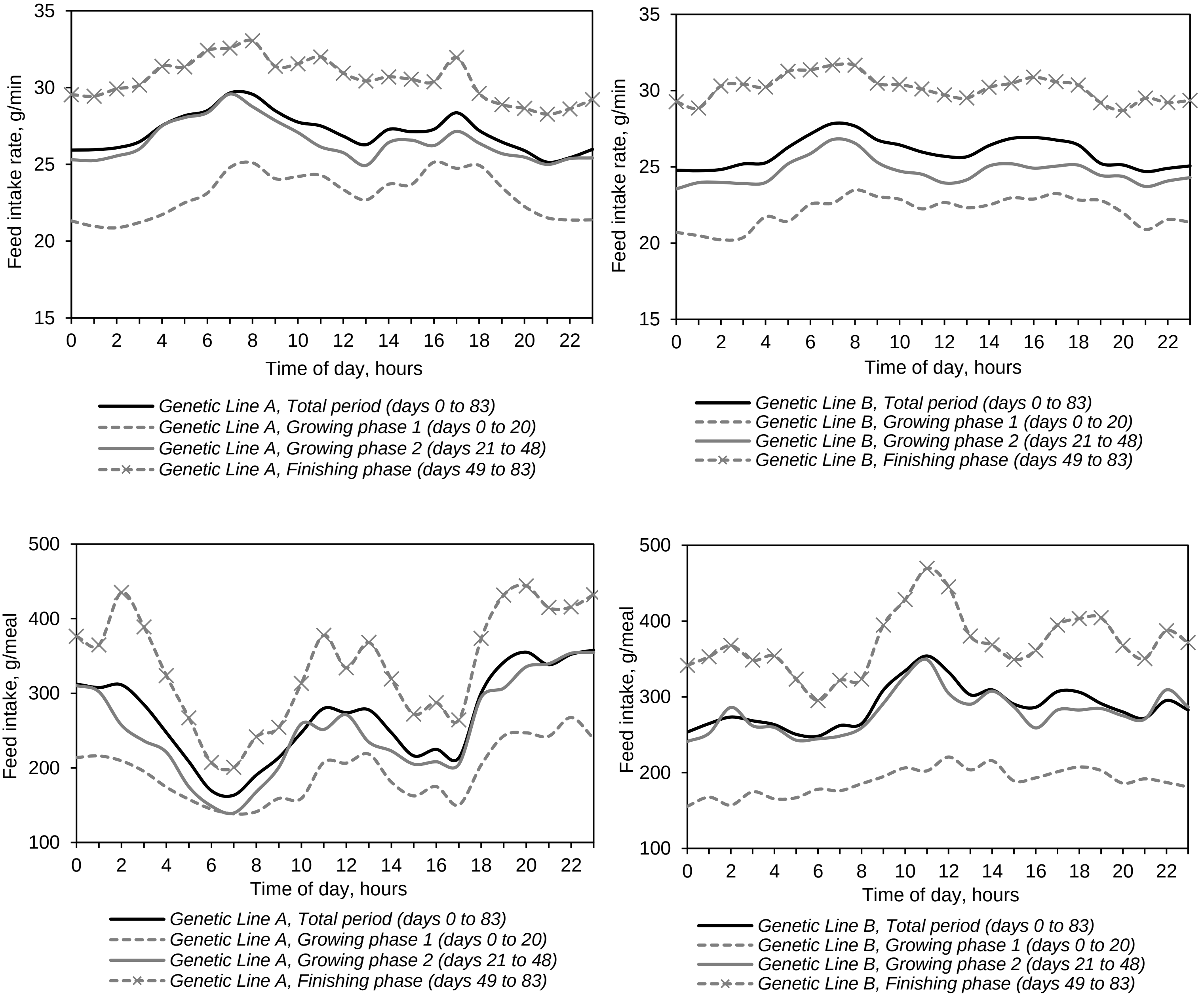

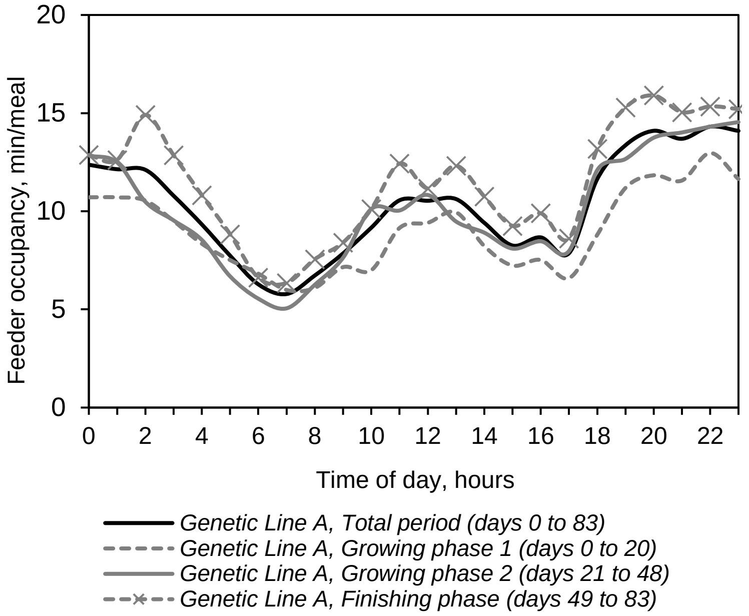
Feeder occupancy, min/meal
Time of day, hours
Genetic Line A, Total period (days 0 to 83)
Genetic Line A, Growing phase 1 (days 0 to 20)
Genetic Line A, Growing phase 2 (days 21 to 48)
Genetic Line A, Finishing phase (days 49 to 83)

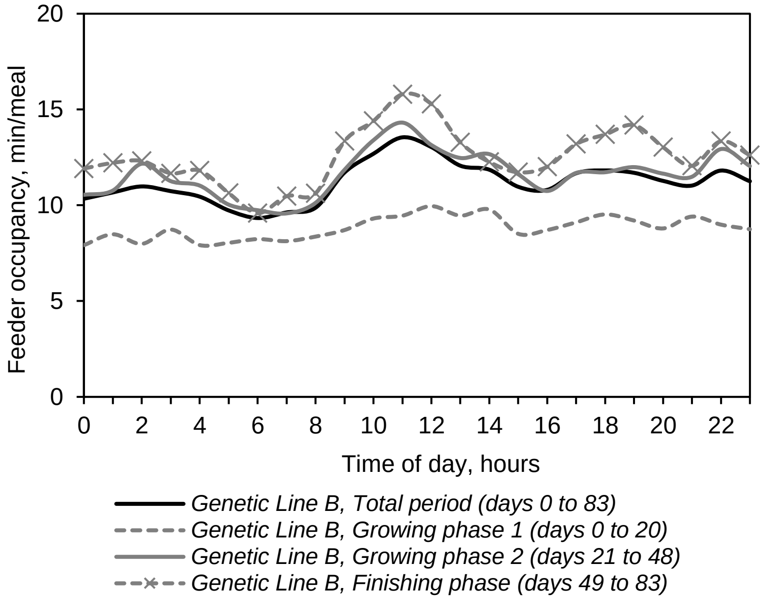
Feeder occupancy, min/meal
Time of day, hours
Genetic Line B, Total period (days 0 to 83)
Genetic Line B, Growing phase 1 (days 0 to 20)
Genetic Line B, Growing phase 2 (days 21 to 48)
Genetic Line B, Finishing phase (days 49 to 83)

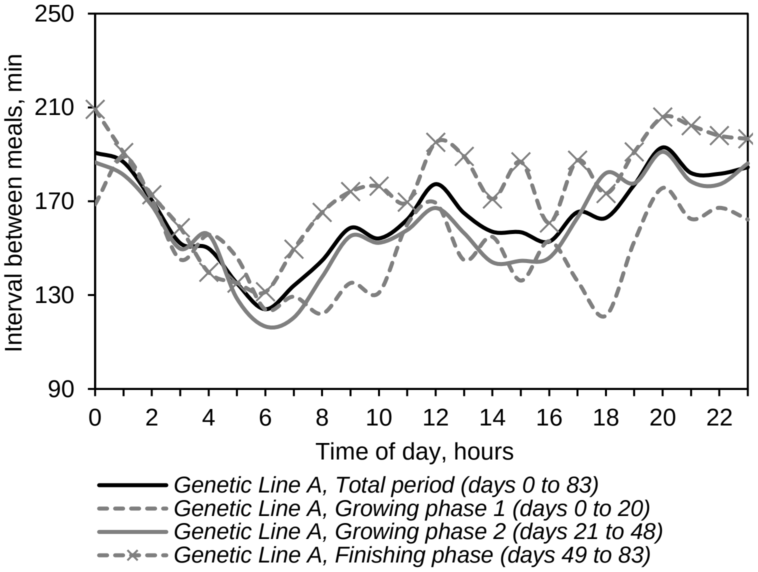
Interval between meals, min
Time of day, hours
Genetic Line A, Total period (days 0 to 83)
Genetic Line A, Growing phase 1 (days 0 to 20)
Genetic Line A, Growing phase 2 (days 21 to 48)
Genetic Line A, Finishing phase (days 49 to 83)

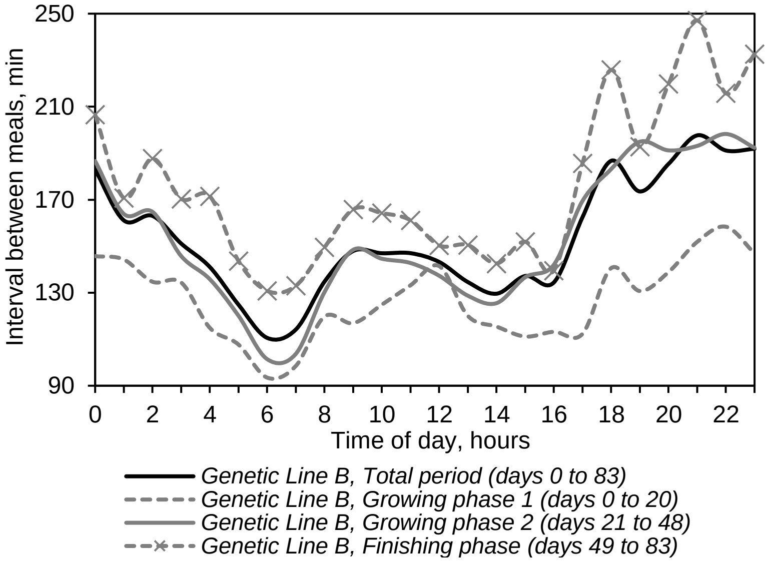
Interval between meals, min
Time of day, hours
Genetic Line B, Total period (days 0 to 83)
Genetic Line B, Growing phase 1 (days 0 to 20)
Genetic Line B, Growing phase 2 (days 21 to 48)
Genetic Line B, Finishing phase (days 49 to 83)

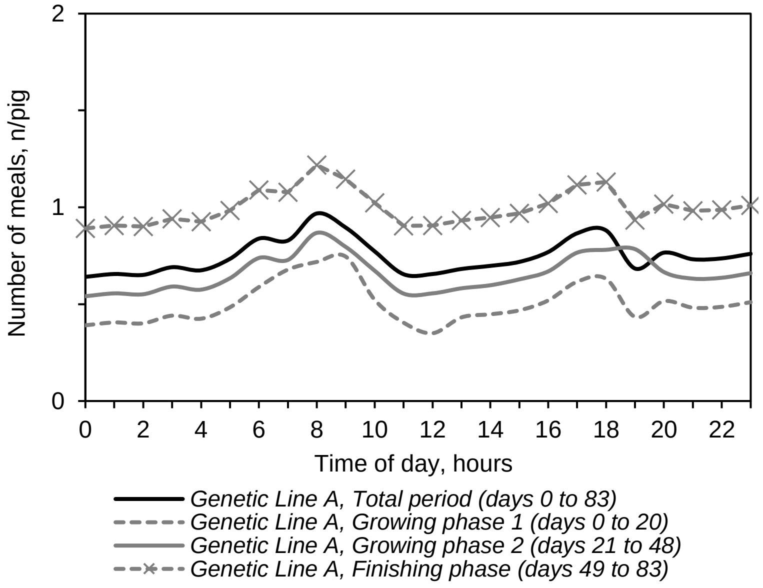
Number of meals, n/pig
Time of day, hours
Genetic Line A, Total period (days 0 to 83)
Genetic Line A, Growing phase 1 (days 0 to 20)
Genetic Line A, Growing phase 2 (days 21 to 48)
Genetic Line A, Finishing phase (days 49 to 83)

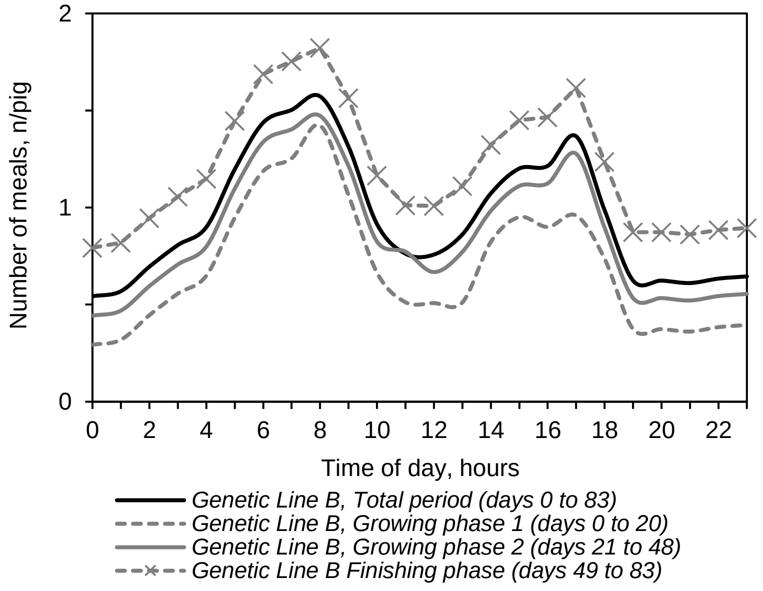
Number of meals, n/pig
Time of day, hours
Genetic Line B, Total period (days 0 to 83)
Genetic Line B, Growing phase 1 (days 0 to 20)
Genetic Line B, Growing phase 2 (days 21 to 48)
Genetic Line B Finishing phase (days 49 to 83)
