# Supplementary material for: Genetic selection modulates feeding behavior of group-housed pigs exposed to daily cyclic high ambient temperatures
Source: PLoS One. 2022 Jan 24;17(1):e0258904. doi: 10.1371/journal.pone.0258904 (PMC8786115; doi:10.1371/journal.pone.0258904)
Supplement: S3 Fig — (DOCX) [file pone.0258904.s003.docx]

Supporting Information

**Genetic selection modulates feeding behavior of group-housed pigs exposed to daily cyclic high ambient temperatures**

Alícia Zem Fraga^1,2^, Luciano Hauschild^1^, Paulo Henrique Reis Furtado Campos^3^, Marcio Valk^4^, Débora Zava Bello^4^, Marcos Kipper^5^, Ines Andretta^5*^

^1^ Department of Animal Science, São Paulo State University, School of Agricultural and Veterinarian Sciences, Jaboticabal, São Paulo, Brazil

^2^ PEGASE, INRAE, Institut Agro, Saint Gilles, France

^3^ Department of Animal Science, Universidade Federal de Viçosa, Viçosa, Minas Gerais, Brazil

^4^ Department of Statistics, Universidade Federal do Rio Grande do Sul, Porto Alegre, Rio Grande do Sul, Brazil

^5^ Department of Animal Science, Universidade Federal do Rio Grande do Sul, Porto Alegre, Rio Grande do Sul, Brazil

*Corresponding Author

Email: [ines.andretta@ufrgs.br](mailto:ines.andretta@ufrgs.br)


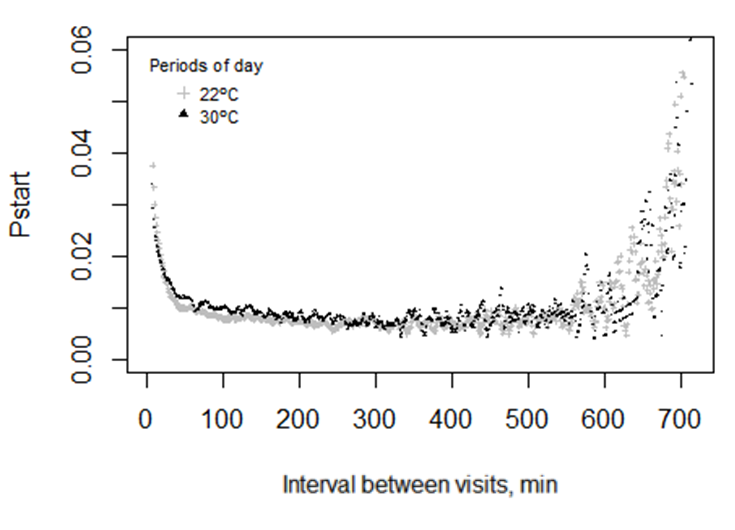


S1 Fig. Probability of growing-finishing pigs exposed to daily cyclic high ambient temperature starting a new feeding event within the next minute since the last visit (Pstart) at 22°C and 30°C.
