# Supplementary material for: Genetic selection modulates feeding behavior of group-housed pigs exposed to daily cyclic high ambient temperatures
Source: PLoS One. 2022 Jan 24;17(1):e0258904. doi: 10.1371/journal.pone.0258904 (PMC8786115; doi:10.1371/journal.pone.0258904)
Supplement: S1 Dataset — (PDF) [file pone.0258904.s004.pdf]

S1 Dataset. Data filtering diagram.

Criteria used for data filtering (see "*Overview of data compilation*" topic).

Days of data record:

18 days of adaptation + 84 days of experimental period

Excluded[1]: 30 days of data record

- Adaptation period[2]: 18 days
- Animal scanner[3]: 3 days
- Animal weighing[3]: 4 days
- Equipment malfunctions[4]: 2 days
- Momentaneous data error record[4]: 3 days

[1] Such days were excluded from the database because they were considered without interest for this study (atypical conditions).
[2] Animals adaptation period to automatic feeders.
[3] For more details see our previously publication Fraga et al., 2019 (doi: 10.1093/jas/skz123).
[4] Such data was likely generated by a technical system failure. However, this was considered a one-off limitation, given that a fairly limited number of experiment days were removed from the dataset.

Initial database: 72 days

Assessed for eligibility (n = 129,167 records)

<u>First data filtering:</u> Interval between visits greater than 720 min (4,883 records were excluded = 3.78% of observations from the initial database).

From the total of deleted records (3.78%):

- 58.0% were from Genetic Line A pigs
- 42.0% were from Genetic Line B pigs

Data "interval between visits" summary		
	Before data filtering	After data filtering
Minimum	0.02	0.02
First quartile	3.17	1.13
Median	10.14	7.05
Mean	53.70	50.52
Third quartile	310.18	54.15
Maximum	992.30	719.45

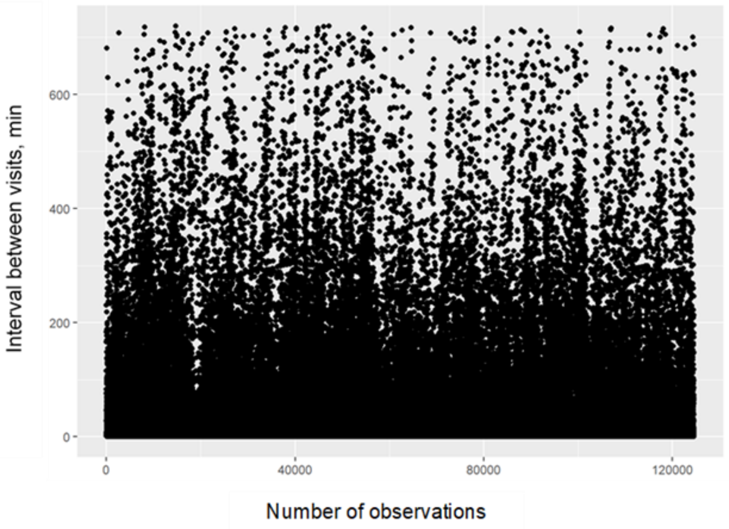

Figure 1. Interval between visits distribution after data filtering.

<u>Second data filtering:</u> Feeder occupancy greater than 30 min (597 records were excluded = 0.48% of observations from the database generate after the first data filtering).

From the total of deleted records (0.48%):

- ♦ 51.8% were from Genetic Line A pigs
- ♦ 48.2% were from Genetic Line B pigs

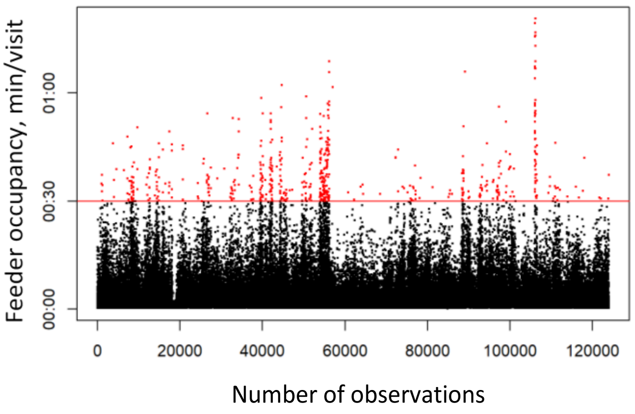

Figure 2. Feeder occupancy distribution before data filtering.

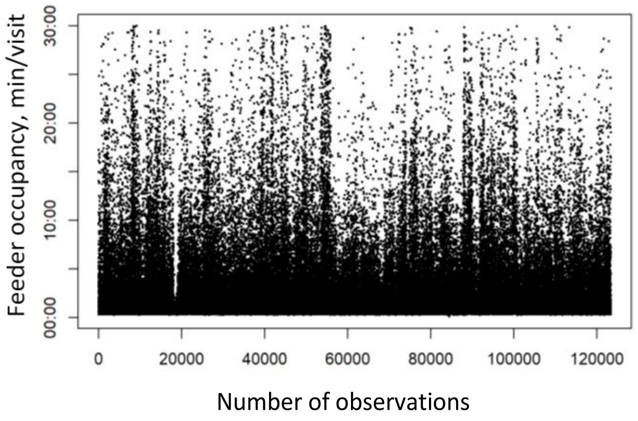

Figure 3. Feeder occupancy distribution after data filtering.

<u>Third data filtering:</u> Data records with zero feed intake (433 records were excluded = 0.35% of observations from the database generate after the second data filtering).

From the total of deleted records (0.48%):

- ♦ 61.2% were from Genetic Line A pigs
- ♦ 38.8% were from Genetic Line B pigs

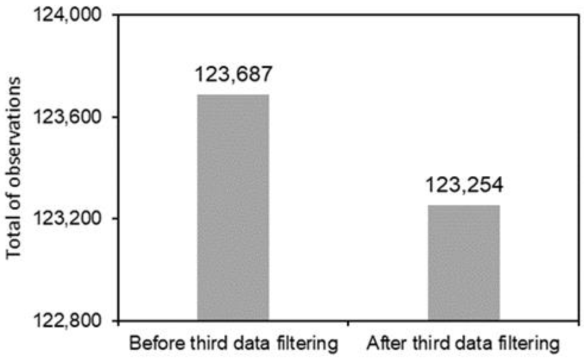

Figure 4. Total of observation before and after third data filtering.

Final database

n = 123,254 records

Database used to calculate meal criteria
